# Cochlear Implant Surgery: Endomeatal Approach versus Posterior Tympanotomy

**DOI:** 10.3390/ijerph17124187

**Published:** 2020-06-12

**Authors:** Francesco Freni, Francesco Gazia, Victor Slavutsky, Enrique Perello Scherdel, Luis Nicenboim, Rodrigo Posada, Daniele Portelli, Bruno Galletti, Francesco Galletti

**Affiliations:** 1 Department of Adult and Development Age Human Pathology “Gaetano Barresi”, Unit of Otorhinolaryngology, University of Messina, 98125 Messina, Italy; franco.freni@tiscali.it (F.F.); danieleportelli@yahoo.it (D.P.); bgalletti@unime.it (B.G.); fgalletti@unime.it (F.G.); 2 Ent Clinic, 08001 Barcelona, Spain; victor.slavutsky@gmail.com; 3 Servicio de ORL, Hospital Universitario Vall d’Hebron, 08001 Barcelona, Spain; 8929eps@comb.cat; 4 Ear Institute, Universidad Abierta Interamericana, Rosario S2000, Argentina; entgazia@gmail.com; 5 Servicio de ORL, Universidad Tecnológica de Pereira, Pereira 660001, Colombia; aagazia@gmail.com

**Keywords:** endomeatal approach, cochlear implant, hearing loss, posterior tympanotomy, tinnitus, without mastoidectomy

## Abstract

The aim of the present study was to compare the posterior tympanotomy (PT) technique to the endomeatal approach. The endomeatal approach (EMA) for Cochlear Implant (CI) surgery was performed on 98 patients with procident lateral sinus or a small mastoid cavity, on 103 ears (Group A). Conventional mastoidectomy and PT was performed on the other 104 patients, on 107 ears (Group B). Data on all patients were then collected for the following: intra- and post-operative complications, Tinnitus Handicap Inventory (THI), Vertigo Symptom Scale (VSS), duration of surgery, and postoperative discomfort. The difference in the total number of major and minor complications between the case group and the control group was not statistically significant. There was a statistically significant difference in discomfort between the two groups using the Visual Analogue Scale (VAS), both immediately postsurgery (*p* = 0.02) and after one month (*p* = 0.04). The mean duration of surgery was 102 ± 29 min for EMA and 118 ± 15 min for the PT technique (*p* = 0.008). EMA is a faster technique resulting in reduced postoperative patient discomfort in comparison to the PT method. The experience of the surgeon as well as the correct choice of surgical technique are fundamental to successful outcomes for cochlear implant surgery.

## 1. Introduction

Cochlear Implant (CI) surgery is now the most common and reliable method used to treat patients with severe and profound sensorineural hearing loss. A CI is a surgically implanted medical device that converts acoustic sound input into electrical stimuli. The acoustic information is manipulated by the CI’s speech processor to generate electrical signals, which directly stimulate the auditory nerve. Hair cells in the inner ear are not involved in the process [1,2,3,4].

The traditional surgical procedure (the transmastoid approach) involves an antromastoidectomy, followed by posterior tympanotomy (PT) through the facial recess and finally a round window technique or promontorial cochleostomy. This surgical approach has been used routinely for several years with very satisfactory results if performed by experienced otological surgeons. The main difficulty with this approach is the risk of facial nerve damage [5,6,7,8]. Alternative techniques (nonmastoidectomy approaches) have been developed in recent years. These are particularly useful when anatomic constraints are present and mean that a facial recess approach is difficult to perform. In these cases, alternative techniques must be used to minimize complications. One of the nonmastoidectomy approaches in CI surgery is the endomeatal approach (EMA). This allows an optimal and atraumatic insertion plane for the positioning of the array through the external auditory canal (EAC) and the round window (RW) [9,10].

A surgical approach using the external auditory canal and the round window as a natural access pathway for cochlear implant positioning, the endomeatal approach, is described. This approach avoids performing an antromastoidectomy, the subsequent posterior tympanotomy, and the promontorial cochleostomy. EMA requires making a bony EAC groove for electrode lead lodging in order to avoid contact between the skin and the EL that could lead to its extrusion. An overhang is left in the superior groove’s edge in order to retain the electrode lead and avoid its contact with the EAC skin, therefore preventing extrusion. A 1 mm width and 2 mm depth is enough to cross the fallopian canal at a safe distance and lodge the electrode.

There are several differences between EMA and the traditional PT approach.

During the PT technique, the RW and the promontory are accessed from the back after the mastoidectomy is carried out. During the EMA procedure, the RW is accessed from a different point of view and located through the front of the posterior wall of the EAC.

A better insertion angle is obtained for EMA in comparison to PT because the scala tympani is in the same line of the insertion plane, meaning that the array does not hit the spiral lamina during introductory maneuvers. The insertion angle is approximately 30° more anterior and 15° more superior in comparison to the PT insertion angle and follows the longitudinal axis of the scala tympani. Ruptures of the modiolar wall, spiral lamina, and/or basilar membrane are avoided during EMA because the insertion line is in a more vertical position, meaning that there is enough space in the EAC to allow the array to curve over the scala tympani wall [11]. When using the traditional PT approach, there is a risk of facial nerve lesion during the mastoidectomy and PT. The incidence of this condition is around 1%. Anomalies in the facial nerve course are present in 16% of patients with CI, and one third of these have either common cavity malformations or hypoplastic cochleas with aberrant facial nerve courses [9].

The groove created during EMA enables the risk of facial nerve damage to be reduced, because the groove is in the posterior EAC wall, away from the nerve in a position easily controlled through the visualization of the Fallopian canal over the oval window.

PT through the facial recess should require facial nerve monitoring. Furthermore, the chorda tympani (CT) is often not preserved when drilling the bone, but is sometimes intentionally sacrificed in order for the surgeon to obtain a better view of the round window. During EMA, the CT is easily visualized and preserved [12].

A cholesteatoma may also develop when a PT is performed, caused by penetration of the skin in the mastoid cavity via the hole left in the posterior EAC wall. The EMA approach avoids this complication [10].

The aim of the present study was to compare the traditional transmastoid technique with EMA to determine any statistically significant differences between the two techniques for the following: intra- and postoperative complications, tinnitus, vertigo, postsurgery discomfort, and duration of intervention.

## 2. Materials and Methods 

A retrospective multicenter study was carried out in the Department of Otorhinolaryngology at Policlinic G. Martino of Messina in Italy, the Instituto del Oído of Rosario in Argentina, and the University of Pereira in Columbia from 2005 to 2018. In total, 202 patients with deafness who had received cochlear implant surgery were enrolled, with 210 implanted ears. We included in our study all adult patients aged over 18 years with bilateral sensorineural hearing loss, who did not benefit from hearing aids, meeting at least one of these criteria:

1. A pure tone average (PTA) at 0.5, 1, 2, and 4 kHz of worse than 55 dB when tested using pure tone audiometry with bilateral hearing aids.

2. A word recognition score (WRS) of <50% recognition of bisyllabic words in open lists with optimized bilateral hearing aids.

The exclusion criteria were as follows: patients who had received previous middle ear surgery, and patients with cochlear otosclerosis or other significant craniofacial malformations. Altogether, 98 patients with procident lateral sinus, a small mastoid cavity or a narrow facial recess received the endomeatal approach (Group A), with 103 ears. An additional 104 patients with no anatomic variations, with 107 ears, received conventional mastoidectomy and TP (Group B) (Table 1). All groups received the following: intraoperative Neuronal Response Telemetry, five days of broad-spectrum antibiotics by injection, and a Stenvers projection X-ray of the skull to verify the correct positioning of the inner part of the CI the day after surgery. Activation and mapping was performed one month after surgery. We also carried out follow up assessments at 1, 3, 6, and 12 months postsurgery and once a year for the rest of life. All patients were assessed for the following: intra- and postoperative complications; Tinnitus Handicap Inventory (THI) value; Vertigo Symptom Scale (VSS) value; duration of surgery; and postoperative discomfort.

### 2.1. Surgical Technique

In recent times, the endomeatal approach (EMA) has been introduced for CI surgery. EMA avoids the need for mastoidectomy and PT by using the external auditory canal (EAC) and the round window (RW) as a natural access pathway for CI positioning. The surgery is carried out using the following steps:

-Incision of the skin of the EAC followed by detachment and overturn of the tympanomeatal flap.

-Prolonged retroauricular skin incision in an S-shaped cephalic direction.

-Retroauricular detachment of the skin of the posterior wall of the external third of the EAC.

-Identification of the RW, removal of the overhanging bone projection that protects the RW until complete exposure of the RW membrane.

-Formation of a bone canal 2 mm from the tympanic groove for the RW approach, proceeding towards the outer edge of the bone of the EAC between its posterior and superior wall until it reaches the temporal squama.

-An overhang is left in the superior groove’s edge in order to house the electrode lead and avoid it making any contact with the skin of the EAC, thereby preventing its extrusion.

-The array is inserted into the scala tympani of the RW, following incision into the secondary tympanic membrane.

-The electrode holder filter is placed in the groove previously excavated in the EAC and stabilized with bone powder and fibrin glue.

-The body of the CI is placed in the subperiosteal pocket and secured with a titanium screw [10] (Video S1).

The traditional surgical procedure that we performed (the transmastoid approach) involves an antromastoidectomy, followed by posterior tympanotomy (PT) through the facial recess and finally a round window technique.

Both techniques have been performed by surgeons with at least 20 years of experience.

### 2.2. Intra- and Postoperative Complications

Intra- and postoperative complications were divided into major and minor.

Major complications included array extrusion, necrosis or severe flap infection, facial paralysis, IC failure, biofilm formation, meningitis, implant extrusion, incorrect positioning of the array, and liquorrhea from the fixing holes, requiring new intervention or causing permanent disability.

Minor complications included mild infection of the flap, hematoma, lesion of the chorda tympani with taste disturbances, selective stimulation of the facial nerve, dizziness and postoperative balance disorders, postoperative tinnitus, and perilymphatic fistula managed with drug therapy or local surgery.

### 2.3. Tinnitus Handicap Inventory

The Tinnitus Handicap Inventory (THI) is a self-administered test used to determine the degree of distress suffered by the patient with tinnitus. It consists of 25 questions divided into three subgroups: functional, emotional, and catastrophic. Eleven items are included on the functional scale, nine on the emotional scale, and five on the catastrophic scale. The THI uses a three-point scale: 0 (No), 2 (Sometimes), and 4 (Yes). The total score ranges from 0 to 100, and a higher score indicates a higher frequency of symptoms [13].

### 2.4. Vertigo Symptom Scale-Short Form

The Vertigo Symptom Scale-short form (VSS-sf) is a fifteen-item, self-administered assessment that measures the frequency of vertigo, dizziness, unsteadiness, and concomitant autonomic/anxiety symptoms over the past month. The VSS-sf uses a five-point Likert scale: 0 (never), 1 (a few times), 2 (several times), 3 (quite often/every week), and 4 (very often/most days). The total score ranges from 0 to 60, and a higher score indicates a higher frequency of symptoms [14].

### 2.5. Discomfort Visual Analogue Scale

The Discomfort Visual Analogue Scale (VAS) is a self-administered assessment that measures the discomfort of patients postsurgery (including pain, headache, constriction, etc.). The total score ranges from 0 to 10, and a higher score indicates a higher frequency of symptoms.

### 2.6. Ethical Approval 

All procedures performed in studies involving human participants were in accordance with the ethical standards of the institutional and/or national research committee and with the 1964 Helsinki declaration and its later amendments or comparable ethical standards. The study was approved by the Ethics Committee of the University of Messina, on 08/27/2019 with the protocol number 81/19.

### 2.7. Statistical Analysis

Statistical analyses were performed using SPSS 25.0 (IBM SPSS Statistics, New York, NY, USA). The data are presented as means with standard deviations. Data normality was assessed using the Kolmogorov–Smirnov test of normality. The Mann–Whitney U-Test was used to compare the THI, VSS-sf, and Discomfort VAS measurements between the groups. Student’s *t*-test was used to compare the PTA, WRS, months of follow-up, and ages of the groups. An ANOVA test with a post-hoc Tukey’s HSD Test was used to compare the THI, VSS-sf, and Discomfort VAS follow-up values of each group. The chi-square test was used to compare the gender distribution of the groups as well as the etiology of deafness. The Fisher exact test was used to determine the percentage of complications that arose in each group and the implant model. Odds ratios and their corresponding 95 % Confidence Intervals (CIs) were calculated. A *p* ≤ 0.05 was considered to be significant.

## 3. Results

Following the application of inclusion and exclusion criteria, 98 patients, of whom 52 were male and 46 were female, with a mean age of 54.35 ± 11.34, were selected for the A group. A total of 104 patients, of which 60 were male and 44 were female, with a mean age of 51.72 ± 8.11 were selected for the B group. There were no statistically significant differences between the two groups for the following: age, gender, etiology of deafness, model of implants, PTA and WRS values with aids, THI, and VSS-sf values presurgery and at follow-up.

There was only one major complication in group A, an ear infection with a biofilm formation that required CI removal, and no statistically significant difference between the two groups for this variable (*p* = 0.45, Odds Ratio = 3.1, 95% CI = 0.1–78.1). For minor complications, there were three cases of tympanic perforation in group A, and cases of subcutaneous emphysema, partial migration of the implant body, and facial nerve stimulation in group B. There was no statistically significant difference between the two groups for minor complications. The total number of minor complications was also not found to be statistically significant when comparing the case group to the control group (*p* = 1, Odds Ratio = 1.1, 95% CI = 0.2–5.2) (Table 2).

THI, VSS-sf, and Discomfort VAS values were calculated postsurgery and after 1, 3, 6, and 12 months (Table 3). There was an improvement in THI postsurgery in comparison to presurgery values (*p* = 0.007), with a further reduction in handicap after each follow-up in each of the groups (*p* < 0.001). There was no statistically significant difference between the two groups for THI scores.

There was an increase in vertiginous sensation immediately after the operation recorded on the VSS-sf, in comparison to the basal condition (*p* < 0.001). During follow-up, there was a clear improvement in these symptoms (*p* < 0.001). There was no statistically significant difference between the two groups for VSS-sf scores.

A statistically significant difference was found between the two groups for Discomfort VAS values postsurgery (*p* = 0.02) and after 1 month (*p* = 0.04). Subsequent follow-ups did not show significant differences. The mean duration of surgery was 102 ± 29 min for EMA and 118 ± 15 min for the traditional technique, with a statistically significant difference between them (*p* = 0.008).

## 4. Discussion

The endomeatal approach (EMA) is a nonmastoidectomy approach in which cochlear implant positioning is carried out through the external auditory canal and the round window.

Our study demonstrates that no statistically significant differences were present in intra- and postoperative complications, or in the presence of tinnitus and vertigo postsurgery between groups A and B. A higher discomfort was recorded in Group B after the surgery, and a month later, whereas no statistically significant differences were present at 3, 6, and 12 months follow-up. Hospitalization time does not change between the two techniques. On average, a patient remains hospitalized for 3–4 days. This shows that both techniques are valid. The surgeon’s experience with carrying out both techniques is fundamental for the success of the intervention. It is therefore advisable to evaluate on a case-by-case basis as to which of the two techniques might yield the best result.

A recent review of the literature suggests that anatomical variability is an important factor in cochlear implant surgery and is usually present [9]. The anatomy of the ear is very complex and an in-depth knowledge of this is required before carrying out surgery. The traditional approach with PT becomes more difficult in patients with procident lateral sinuses and small mastoid cavities, both of which are diagnosed with some frequency, as the surgical space for insertion of the array is reduced. With the EMA technique, insertion is safe and easy [15].

Craniofacial malformations are often present in syndromic children with congenital deafness, making traditional CI surgery with mastoidectomy and PT difficult. In the literature, EMA is also used for children with minor anatomical variations. As the child grows, there is the potential risk of dislocation of the position of the implant. The groove may then be transformed into a small furrow, which allows the passage of the guide electrode to be housed in a small mastoid fossa of about 2 cm wide, by 2 mm deep, without opening the antromastoid, and located immediately in front of the array housing. This small pit allows placement of the excess cable of the implant. Further studies on the use of EMA in children are necessary [16,17].

EMA is a less invasive technique that facilitates cochlear implant surgery in cases of anatomic differences, such as procident lateral sinus, small mastoid cavity, narrow facial recess, and an anteriorly located facial nerve.

For Tarabichi et al., there was significant variability in the relationship between the ear canal and the basal turn of the cochlea in reference to the sagittal plane. A clear majority of images demonstrated the basal turn of the cochlea to align with a more posterior angle than that of the ear canal. The trajectory provided by posterior tympanotomy aligns more favorably with the basal turn of the cochlea than transcanal access. Endoscopic technique, primarily an ear canal intervention, may not be useful in cochlear implant surgery [18].

Zernotti et al. performed a multicenter review of 208 patients with cochlear implants, comparing the different techniques. The complications were classified into major and minor. Among the 208 implanted patients, 10.5% (22 of 208) had complications. Of these, 2.88% (6 of 208) were major complications and 7.69% (16 of 208) were minor complications. Comparing the results obtained by the different approaches, the PT technique had the lowest rate of major complications (1.1%), followed by the EMA technique with 2.38% and suprameatal approach with 3.75%. As for minor complications, operations in the suprameatal approach group had the lowest rate (6.25%), followed by the EMA group (7.14%), and the group operated on using the PT technique presented the highest (10%) [19].

Mostafa et al. performed a prospective study on 125 cochlear implant patients and followed up for 6–30 months. A modified transcanal technique was adopted through a small postauricular incision. A tympanomeatal flap is elevated, the middle ear is exposed, and the round window membrane is exposed by drilling the overhanging niche. The electrode is channeled in an open trough along the posterosuperior meatal wall, which is reconstructed by autologous cartilage. The round window was used for insertion in 110 patients and a cochleostomy in 15. There were 115 complete insertions and 10 partials. There were six chorda tympani injuries, two electrode exposures with one requiring revision, and two cases with a tympanic membrane perforation which were grafted uneventfully. One case had severe infection and subsequent extrusion of the device one year after successful implantation [4].

Thaiba et al. reported the transmeatal approach that involves creating an open transcanal tunnel starting from the annulus superior to the chorda tympani laterally towards the suprameatal region. Then, through the open tunnel, a bony groove is created in the bone underneath the length of the EAC to protect the electrode from extrusion through the EAC. They described the use of this approach in 131 patients. During 2 to 46 months of follow-up, there was no electrode extrusion [20].

A shorter duration of surgery was obtained for EMA in comparison to the traditional technique, as observed by Slavutsky et al. [10,21].

A limitation of our study is that it is retrospective, with the choice of surgical technique being based on anatomical radiodiagnostic study. The study was conducted over a long period of time and in three different countries. Many surgeons were involved in the EMA and PT technique, and the medical care systems were different in each country, increasing the risk of bias. Given the limitations, our manuscript should be considered as a preliminary study. For significant results, prospective randomized double or triple-blind studies are required. Cochlear implant surgery is very delicate, regardless of the surgical technique chosen, meaning that the surgeon must have strong expertise and have mastered both techniques. Accurate presurgical planning, with careful analysis of radiological studies, is necessary when choosing the best surgical technique for each case, in order to obtain the best results from intervention.

## 5. Conclusions

Our multicenter study confirms that EMA is a safe surgical technique with excellent outcomes. There were no statistically significant differences with regard to complications. EMA, in our study, appears to be a faster technique with reduced postoperative patient discomfort in comparison to the more traditional PT technique. In each case, the experience of the surgeon and the correct choice of technique are fundamental in achieving a successful outcome for CI surgery.

## Figures and Tables

**Table 1:** Study population.

Features	Group A (EMA) *n* (%) M ± SD	Group B (PT) *n* (%) M ± SD	*p*-Value	Odds Ratio (95% CI)
Gender			0.50	0.82 (0.47–1.44)
Male	52/98 (53%)	60/104 (57.7%)		
Female	46/98 (47%)	44/104 (42.3%)		
Age (years)	54.35 ± 11.34	51.72 ± 8.11	0.25	
Deafness Etiology				
Genetic	50/98 (51%)	65/104 (62.5%)	0.10	0.62 (0.35–1.1)
Autoimmune	10/98 (10.2%)	13/104 (8%)	0.61	0.79 (0.33–1.9)
Infection	15/98 (15.3%)	12/104 (11.5%)	0.43	1.3 (0.61–3.1)
Idiopathic	23/98 (23.4%)	14/104 (13.4%)	0.07	1.9 (0.94–4.01)
Implant model				
Med-El	2/103 (1.9%)	3/107 (2.8%)	0.99	0.68 (0.11–4.19)
Advanced Bionics	4/103 (3.8%)	4/107 (3.7%)	0.99	1.04 (0.25–4.27)
Oticon Medical	1/103 (0.9%)	5/107 (4.6%)	0.21	0.2 (0.02–1.74)
Cochlear	96/103 (93.4%)	95/107 (88.9%)	0.33	1.73 (0.65–4.58)
PTA with aids (dB)	62.25 ± 5.30	59.84 ± 4.26	0.28	
WRS with aids (%)	60.5 ± 3.25	58.45 ± 4.65	0.35	
VSS presurgery	0.75 ± 0.28	0.88 ± 0.37	0.65	
THI presurgery	35.25 ± 10.32	38.15 ± 9.65	0.67	
Follow-up (months)	84.35 ± 15.66	82.75 ± 12.4	0.55	

*n*—number; %—percentage; M—media; SD—standard deviation; CI—confidence interval; PTA—Pure Tone Average; WRS—Word Recognition Score; THI—Tinnitus Handicap Inventory; VS—Vertigo Symptom Scale.

**Table 2:** Intra- and postoperative complications.

Complications	Group A (EMA) *n* (%)	Group B (PT) *n* (%)	*p*-Value	Odds Ratio (95% CI)
Major Complications	1 (0.9%)	0	0.45	3.1 (0.1–78.1)
Bacteria Biofilm	1 (0.9%)	0	0.45	3.1 (0.1–78.1)
Others	0	0		
Minor Complications	3 (2.9%)	3 (2.8%)	1	1.1 (0.2–5.2)
Tympanic perforation	3 (2.9%)	0	0.11	7.4 (0.3–107.7)
Subcutaneous emphysema	0	1 (0.9%)	1	0.3 (0.01–8.5)
Partial migration of the implant body	0	1 (0.9%)	1	0.3 (0.01–8.5)
Facial nerve stimulation	0	1 (0.9%)	1	0.3 (0.01–8.5)
Others	0	0		

*n*—number; %—percentage; CI—confidence interval

**Table 3:** THI, VSS-sf, and VAS discomfort postsurgery and after 1, 3, 6, and 12-month follow-up.

Follow-Up	Group A (EMA) M ± DS	Group B (PT) M ± DS	*p*-Value
THI			
After surgery	11.8 ± 7.2	13.3 ± 8.1	0.45
1 month	9.6 ± 5.4	8.9 ± 4.6	0.55
3 months	5.4 ± 2.3	6.6 ± 2.1	0.59
6 months	2.9 ± 0.9	3.4 ± 1.2	0.21
12 months	2.1 ± 0.7	1.6 ± 0.3	0.23
VSS-sf			
After surgery	21.8 ± 9.4	19.7 ± 8.3	0.22
1 month	4.3 ± 1.2	5.8 ± 1.7	0.19
3 months	1.2 ± 0.2	1.7 ± 0.3	0.65
6 months	0.1 ± 0.02	0.1 ± 0.02	0.93
12 months	0	0	1
Discomfort VAS			
After surgery	3.2 ± 1.1	5.3 ± 1.3	**0.02**
1 month	1.9 ± 0.4	3.2 ± 0.8	**0.04**
3 months	0.2 ± 0.05	0.5 ± 0.06	0.12
6 months	0.01 ± 0.002	0.01 ± 0.002	0.95
12 months	0	0	1

M—media; SD—standard deviation; THI—Tinnitus Handicap Inventory; VSS-sf—Vertigo Symptom Scale short form; VAS—Visual Analogue Scale.

## Supplementary Materials

The following are available online at https://www.mdpi.com/1660-4601/17/12/4187/s1, Video S1: Endomeatal Approach.
